# Enhanced protocol for measuring glutathione peroxidase activity using a new glutathione peroxidase-Tiron assay

**DOI:** 10.1093/biomethods/bpaf075

**Published:** 2025-10-17

**Authors:** Mahmoud Hussein Hadwan, Abbas Ali Mohammed, Saeed Najavand, Roaa Altaee, Asad M Hadwan

**Affiliations:** Department of Chemistry, College of Science, University of Babylon, Hilla City 51001, Iraq; Department of Chemistry, Faculty of Basic Sciences, Azarbaijan Shahid Madani University, Tabriz 53714-161, Iran; Department of Chemistry, Faculty of Basic Sciences, Azarbaijan Shahid Madani University, Tabriz 53714-161, Iran; College of Pharmacy, Al-Zahraa University for Women, Karbala Governorate 11118, Iraq; College of Dentistry, University of Manara, Al-Amarah 12218, Iraq

**Keywords:** glutathione peroxidase, tiron reagent, enzyme activity assay, oxidative stress, clinical diagnostics, spectrophotometry

## Abstract

Conventional methods for measuring glutathione peroxidase (GPx) activity are limited by interference issues, complex protein precipitation steps, and variable reliability, necessitating the development of improved analytical approaches for both research and clinical applications. A modified GPx activity assay has been developed utilizing the Tiron reagent system, which eliminates the need for protein precipitation. The protocol employs a novel termination reagent containing ferrous ion (Fe^2+^) and Tiron (C_6_H_4_Na_2_O_8_S_2_) to instantly stop enzymatic decomposition of hydrogen peroxide. Following GPx-mediated H_2_O_2_ consumption, residual hydrogen peroxide undergoes Fenton-type redox reactions with Fe^2+^ ions, generating Fe³^+^ species that coordinate with Tiron through catechol moieties to form a stable ferri-Tiron complex [Fe(C_6_H_4_Na_2_O_8_S_2_)]³^+^. The assay operates optimally at acidic pH to ensure complex stability and minimize interference from competing reactions. The modified protocol demonstrates superior performance characteristics compared to conventional GPx assays, including elimination of interference effects, enhanced accuracy and precision, and improved reproducibility across diverse sample matrices. The method’s spectrophotometric detection system provides reliable quantification with minimal matrix effects, while the simplified workflow reduces technical complexity and analysis time. This interference-free GPx activity assay offers significant advantages for both laboratory research and clinical diagnostics. It achieves this through a combination of analytical precision, operational simplicity, and broad compatibility with standard laboratory practices and equipment. The protocol’s robust performance at acidic pH conditions, coupled with its elimination of protein precipitation steps, establishes it as a valuable alternative to existing methodologies for assessing oxidative stress and evaluating antioxidant capacity.

## Graphical Abstract

**Figure:**
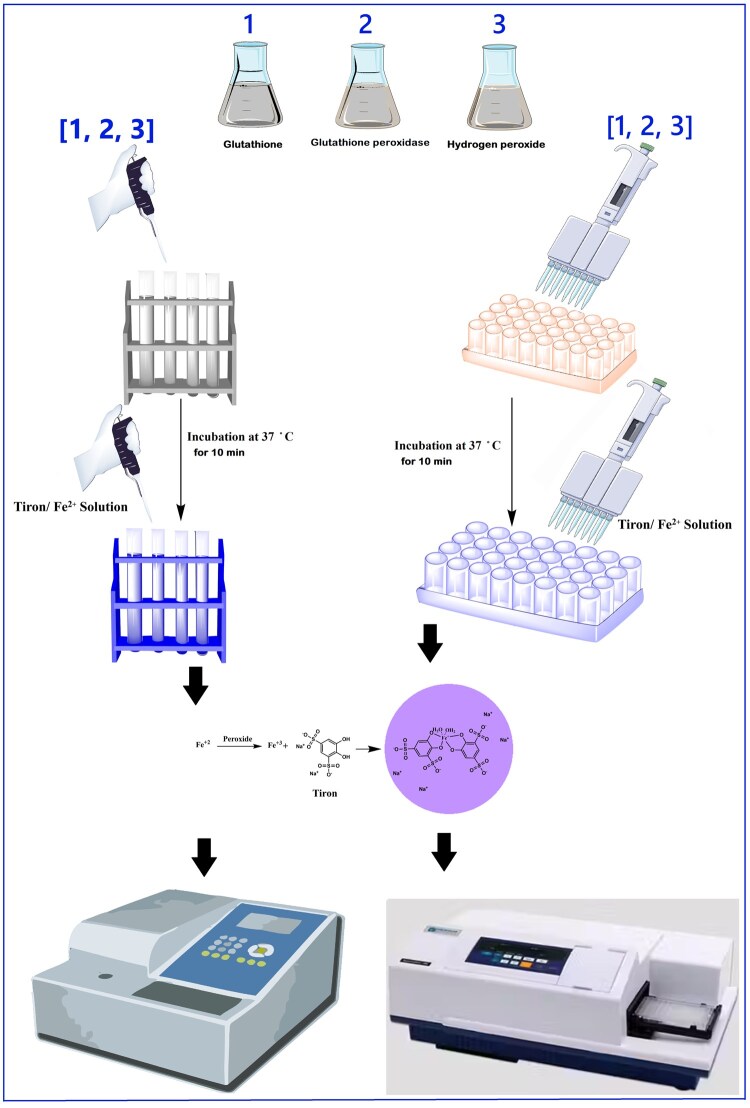

## Background

Glutathione peroxidases (GPxs) are a highly conserved family of oxidoreductase enzymes found in all living organisms, from simple prokaryotes to complex eukaryotes. These enzymes play a crucial role in cellular antioxidant defense systems, serving as primary protectors against damage caused by oxidative stress [1]. G.C. Mills and colleagues first identified the enzyme’s activity they observing the GPx’s ability to shield hemoglobin from oxidative degradation [2]. This vital discovery launched decades of research into the protective mechanisms of the GPx family [3].

During the 1960s, research into GPx expanded as its activity was found in various tissues, including the lungs and kidneys. Scientists have demonstrated that GPx can reduce a range of hydroperoxides, particularly hydrogen peroxide (H_2_O_2_). In the 1970s, a pivotal breakthrough revealed that the enzyme’s activity depended on the presence of selenium, a trace element [4]. This finding clarified that many GPx isoforms are selenium-dependent, leading to the recognition of GPx1 as the most efficient peroxide removal system in mammalian cell cytosol [3].

Glutathione peroxidases are classified under EC 1.11.1.9 and EC 1.11.1.12 and comprise several isozymes that can reduce H_2_O_2_ or organic hydroperoxides (ROOH) to water or alcohols, respectively [5]. The core enzymatic reaction utilizes reduced glutathione (GSH) as an electron donor, as shown in the equation: H_2_O_2_ + 2GSH → GSSG + 2H_2_O. This mechanism is vital for maintaining cellular redox balance, eliminating harmful reactive oxygen species, and regenerating oxidized glutathione disulfide (GSSG) [3, 5].

Since the discovery of GPx1, the mammalian GPx family has grown to include eight distinct isoforms (GPx1–8), each with its tissue distribution, subcellular localization, and specialized function [6]. Among the major selenium-dependent isoforms, GPx1 is broadly distributed and highly abundant in red blood cells, liver, lung, and kidney. It forms a homotetramer and is found in the cytosol, nucleus, and mitochondria, offering widespread protection against oxidative stress. GPx2 is specialized for the gastrointestinal tract, mainly located in the cytosol and nucleus, and protects intestinal epithelial cells from oxidative damage. GPx3, a secreted protein, is widely distributed in organs such as the kidney, lung, and heart, providing extracellular antioxidant protection and also functioning inside cells. GPx4 is unique as a monomer, exhibiting broad substrate specificity and subcellular distribution, and specializes in reducing lipid peroxides and protecting cellular membranes from peroxidative damage [3, 4].

Other specialized isoforms include GPx5 and GPx6, which are closely related to GPx3 but have distinct tissue distributions. GPx5, notably lacking selenocysteine, is secreted in the epididymis, while GPx6 is a selenium-dependent isoform found in the olfactory epithelium of humans and pigs [3]. GPx7, also known as NPGPx, is a recently characterized phospholipid ROOH glutathione peroxidase that contains cysteine instead of selenocysteine. Although it exhibits lower glutathione peroxidase activity *in vitro*, it plays a crucial role in breast cancer cell survival by mitigating oxidative stress resulting from the metabolism of polyunsaturated fatty acids [7].

Beyond their antioxidant function, GPx enzymes are involved in various biological processes, including post-translational protein modifications and cellular signaling [8]. Their redox activity is fundamental to maintaining oxidative homeostasis, with GPX4 standing out for its ability to eliminate lipid peroxides and prevent membrane damage. The evolutionary conservation and functional specialization of GPx isoforms underscore their critical role in cellular defense, ensuring protection across cellular compartments and enabling tailored antioxidant defense in specific tissues [8, 9].

Ongoing research continues to reveal the complex roles of GPx enzymes in health and disease, particularly in their involvement in cancer, neurodegenerative disorders, and the aging process [10]. The identification of selenium-independent isoforms, such as GPx7, has broadened our understanding of the family’s functional diversity and suggests the existence of additional regulatory mechanisms beyond classical selenium-dependent catalysis. Altogether, the GPx enzyme family exemplifies evolutionary adaptation to oxidative stress, with enzymatic diversity and specialization enabling sophisticated cellular protection mechanisms vital for life in an oxygen-rich environment [11].

The measurement of GPx activity has been approached through two primary analytical systems, each offering distinct methodological advantages and limitations. The first system focuses on monitoring substrate consumption by measuring either GSH depletion or ROOH utilization at regular time intervals. The second system employs an indirect approach by coupling GPx activity to glutathione reductase-catalyzed reactions, allowing for the continuous monitoring of GSSG production through measurement of NADPH consumption via spectrophotometric or fluorometric detection [12–14].

The most prevalent approach within the first system utilizes Ellman’s reagent (5,5'-dithiobis-(2-nitrobenzoic acid); DTNB) for colorimetric assessment of GSH consumption as an indicator of GPx activity [15]. Despite its widespread adoption, the GPx-DTNB assay presents several analytical challenges that limit its practical application. The method demonstrates reduced sensitivity compared to alternative protocols and suffers from instability issues with reagents. Additionally, the conventional DTNB-based protocol requires a significant amount of processing time. It relies on intense acid treatment to terminate enzymatic reactions at predetermined intervals, followed by protein precipitation steps that complicate implementation in microplate-based high-throughput systems [16].

Recent methodological improvements have addressed these limitations through the development of a modified DTNB protocol that eliminates the need for protein precipitation while maintaining analytical accuracy [17]. This refined protocol overcomes the traditional disadvantages associated with GPx-DTNB assays, offering improved precision, efficiency, and reliability for both laboratory research and clinical diagnostic applications.

Alternative substrate consumption methods include polarographic techniques developed by Flohé and Günzler [18], which utilize strong acid treatment to halt GPx-catalyzed reactions at specific time points. Following reaction termination, polarographic analysis determines residual GSH content, providing an indirect measure of enzyme activity. While this approach offers analytical precision, it requires specialized polarographic equipment that may limit its accessibility in routine laboratory settings.

Contemporary developments in measuring GPx activity have focused on microplate-based assays utilizing the Cu(II)-neocuproine complex reduction system. In this approach, unreacted GSH reduces Cu(II)-neocuproine to form a highly colored Cu(I)-neocuproine complex, with absorbance changes that inversely correlate with GPx activity [19]. The CUPRAC (Cupric Reducing Antioxidant Capacity) method further refines this process by incorporating catalase to terminate GPx reactions, followed by spectrophotometric detection at 450 nm. However, this method faces significant limitations when applied to biological tissue samples due to interference from endogenous catalase activity, as both GPx and catalase compete for H_2_O_2_, as a common substrate. To overcome these challenges, recent innovations have introduced an interference-free CUPRAC-based method. These involve incubating enzyme samples in phosphate buffer with carefully controlled concentrations of GSH and peroxide substrate [20]. After a defined incubation period, the CUPRAC reagent Cu(Nc)_2_^2+^ is added to stop enzymatic reactions, allowing unreacted substrates to reduce the Cu(II)-neocuproine complex to the intensely colored Cu(I)-neocuproine complex, which exhibits maximum absorbance at 450 nm. In this method, GPx activity is inversely proportional to the decrease in absorbance, providing a robust and reliable analytical framework for accurately measuring GPx activity.

The most widely adopted protocol for GPx activity measurement was established by Paglia and Valentine [21], utilizing the coupled enzyme system approach. This method monitors NADPH consumption at 340 nm as GSSG produced by GPx activity is reduced back to GSH by glutathione reductase. The protocol offers simplicity and selectivity advantages but suffers from poor sensitivity and high reagent costs. Furthermore, NADPH itself functions as a potent GPx inhibitor, potentially compromising assay accuracy [15]. The method’s reliance on UV absorbance measurement presents additional challenges when analyzing biological tissues, as interference from proteins and DNA can compromise analytical precision.

Lawrence and Burk subsequently modified this protocol for specialized applications, including the assessment of GPx activity in liver supernatants from selenium-deficient experimental models [22]. Despite these adaptations, the fundamental limitations regarding sensitivity and potential interference remain problematic for routine clinical applications.

Fluorometric methods represent a significant advancement in the measurement sensitivity of GPx activity. Weiss and colleagues developed a highly sensitive fluorometric method capable of measuring GPx activity in tissue samples as small as 100 µg [23]. This assay exploits the fluorometric properties of NADP^+^ generated from GSSG oxidation during the GPx reaction, providing enhanced sensitivity compared to spectrophotometric methods. Advanced fluorometric techniques employ chemical derivatization strategies to achieve superior analytical sensitivity. Martinez et al. established a fluorometric procedure utilizing o-phthalaldehyde for GSSG detection [24], while Kamata et al. developed an alternative approach using N-(9-acridinyl)maleimide as the fluorescent derivatizing agent [25]. These methods demonstrate exceptional sensitivity and have been successfully applied to the analysis of human plasma samples and liver homogenates, making them particularly valuable for clinical research applications.

The current method presents a modified protocol that addresses the limitations of existing methods for measuring GPx activity. By employing the Tiron reagent, the current method eliminates the need for protein precipitation and resolves the issues previously encountered with the GPx-Tiron assay. The improved method is free from interference, resulting in greater accuracy and reliability. Additionally, it is straightforward to implement in laboratory settings and is well-suited for clinical diagnostics due to its precision, efficiency, and dependability. A key feature of the new protocol is the use of a termination reagent composed of ferrous ion (Fe^2+^) and Tiron (C_6_H_4_Na_2_O_8_S_2_). This reagent immediately inhibits the GPx-mediated decomposition of H_2_O_2_. Once a portion of the original H_2_O_2_ substrate has been consumed by GPx, any remaining H_2_O_2_ reacts with Fe^2+^ ions from the FAS via a Fenton-type redox mechanism, oxidizing Fe^2+^ to Fe³^+^. The newly formed Fe³^+^ ions then coordinate with Tiron molecules through their catechol groups, creating a stable ferri-Tiron [Fe(C_6_H_4_Na_2_O_8_S_2_)]³^+^ complex. This complex exhibits distinct spectrophotometric properties that are applied to measure GPx activity.

The present methodology offers several advantages, including precision, efficiency, and reliability, while remaining interference-free and suitable for both laboratory research and clinical diagnostic applications. The protocol’s simplicity and compatibility with standard laboratory equipment enhance its practical utility across diverse analytical settings.

Table 1 summarizes the principles of the methods employed to measure GPx activity and provides a comparative analysis of their respective advantages and disadvantages.

**Table 1.** The principles, advantages, and disadvantages of methods used to assess GPx activity.

Method	Principle	Advantages	Disadvantages
Classical DTNB (Ellman’s)	Measures GSH depletion via colorimetric DTNB reaction	Simple, widely used, direct measurement of GSH	Lower sensitivity; interference from proteins and matrix components; requires protein precipitation; time-consuming
Coupled GR-NADPH (Paglia & Valentine)	NADPH consumption at 340 nm after GSSG reduction by GR	Simple workflow; continuous measurement; selective	NADPH is a GPx inhibitor, affecting accuracy; poor sensitivity with tissues due to protein/DNA UV absorption; costly reagents
CUPRAC–Cu(II)-Neocuproine	Reduced GSH reacts with Cu(II)-Neocuproine; absorbance at 450 nm	Adaptable to microplate; spectrophotometric readout	Catalase activity interferes; less accurate with biological samples; it requires the addition of catalase to stop the reaction
Fluorometric Methods (e.g. o-phthalaldehyde)	Measures fluorescent derivatives of glutathione	High sensitivity; applicable to small sample volumes	Requires derivatization; more complex instrumentation
Modified Tiron Protocol (current study)	Uses ferrous ion/Tiron termination, spectrophotometric reading at 570 nm	Interference-free; no protein precipitation; accurate, precise, and reproducible; suitable for high-throughput and clinical diagnostics	Requires preparation of fresh reagents and pH control; novel method, less widely adopted

## Procedure

### Chemicals and materials

Tiron, potassium phosphate monobasic (KH_2_PO_4_), sodium acetate (CH_3_COONa·3H_2_O), H_2_O_2_ (30%), sodium azide, sodium acetate, and sodium hydroxide were purchased from Thomas Baker (Chemicals) Pvt Ltd.

Bovine serum albumin, glutathione, Ellman’s reagent DTNB, cumene hydroperoxide (CuOOH), tert-Butyl hydroperoxide (tBuOOH) 70 wt. % in H_2_O, 458139 were purchased from Sigma-Aldrich.

### Buffers and reagent solutions

#### Reagent preparation protocol

##### Reagent 1: Glacial acetic acid solution.

To prepare a 5% (v/v) glacial acetic acid solution, 5 ml of glacial acetic acid was mixed with 95 ml of distilled water.

##### Reagent 2: Tiron solution.

A 9 mM Tiron solution was created by dissolving 299 mg of Tiron in 100 ml of the previously prepared 5% (v/v) glacial acetic acid solution.

##### Reagent 3: Iron(II) chloride solution.

The iron(II) chloride solution was prepared by first dissolving 200 mg of barium chloride in 47.5 ml of distilled water. This solution was then gradually added to a mixture containing 250.218 mg of iron(II) sulfate heptahydrate (FeSO_4_·7H_2_O), which was also dissolved in 47.5 ml of distilled water, while stirring continuously. Afterward, 5 ml of glacial acetic acid was introduced to the mixture. The resulting barium sulfate precipitate was filtered to yield a clear iron(II) solution. This solution was stored in a brown bottle, kept in a dark place, where it remained stable for up to one month, achieving a final concentration of ferrous ions at 9 mM.

##### Reagent 4: Fresh working solution.

A fresh working solution at a concentration of 4.5 mM was prepared by mixing 50 ml of the ferrous (Fe2+) solution with 50 ml of the Tiron solution. The final total volume was adjusted to 100 ml.

### Sodium acetate solution

To prepare a 20% (w/v) sodium acetate (CH_3_COONa·3H_2_O) solution, 20 g of sodium acetate (CH_3_COONa·3H_2_O) was dissolved in 80 ml of distilled water. The final volume was adjusted to 100 ml.

### Protein concentration determination

The protein concentration was assessed using the Bradford Protein Colorimetric Assay Kit (Elabscience; Cat. No.: E-BC-K168-M).

### Phosphate buffer preparation

To produce a 100 mM phosphate buffer solution at pH 7.0, solutions A and B were combined in equal volumes. Solution A was formed by dissolving 13.62 g of KH_2_PO_4_ in 1 l of distilled water, while solution B was made by dissolving 17.8 g of Na_2_HPO_4_·2H_2_O in 1 l of distilled water. The final solution was gently mixed, and 0.372 g, containing 0.6501 g of NaN_3_ and EDTA, was added.

### Reduced glutathione solutions

A 2 mM solution of reduced glutathione was prepared by dissolving 0.1228 g of reduced glutathione in 100 ml of 100 mM phosphate buffer solution (pH 7.0).

### H_2_O_2_ solution

A daily preparation of a 2 mM H_2_O_2_ solution (pH 7.0) was made using the 100 mM phosphate buffer. The final concentration was adjusted according to the molar extinction coefficient of H_2_O_2_, which is 43.6 M^−1 ^cm^−1^ at 240 nm.

### Sodium phosphate solution

A 2% (w/v) sodium phosphate (Na_2_HPO_4_) solution was created by dissolving 2 g of Na_2_HPO_4_ in 80 mL of distilled water, then adjusting the final volume to 100 ml with distilled water.

### tBuOOH preparation

To prepare a 1 M solution of tBuOOH, 130 μl of 70% t-BOOH (approximately 7.7 M) was added to 870 μl of 100 mM phosphate buffer solution (pH 7.0). A 2 mM tBuOOH solution was then prepared by combining 200 μl of the 1 M t-BOOH solution with 99.8 ml of phosphate buffer solution (pH 7.0).

### Blood specimens

Three milliliters of whole blood were collected into a heparinized tube for preparation of erythrocyte lysates. The tubes were centrifuged at 400*g* for 10 min to separate the plasma fraction and buffy coat. The red blood cells were washed three times with 500 μl of 0.9% sodium chloride, after which 500 μl of the erythrocyte suspension was combined with 2 ml of ice-cold double-distilled water. The mixture was vortexed for 10 s and then kept in the dark at 4°C for 15 min. The resulting stock hemolysate was diluted 1:500 and resuspended in 50 mM phosphate-buffered saline; the diluted hemolysate was subsequently used as the source of GPx activity.

### Tissue preparation

Male albino rats and mice were obtained from the Bioscience Department animal house at the University of Babylon (Iraq) for the experimental study. Livers were surgically excised prior to assessment of GPx activity and were thoroughly rinsed with 0.9% (w/v) NaCl to remove blood and contaminants. Each liver was homogenized in cold 1.15% (w/v) KCl using a glass homogenizer, and the homogenate was filtered through two layers of muslin to remove cellular debris. The filtrate was diluted 1:500 with 50 mM phosphate-buffered saline, and the diluted liver homogenate was used for subsequent GPx activity assays.

### Ethics approval

College of Science Ethics Committee, University of Babylon, Iraq; Ref. No. 2155E, dated 27 November 2024.

### The microplate protocol

The details of the procedure are shown in Table 2.

**Table 2.** The details of the protocol used to measure glutathione peroxidase activity.

Reagent	Test (µl)	Standard (µl)	Control (µl)	Blank (µl)
Sample	10		10	
Glutathione (500 µM)	100	100		
D.W		10	100	110
Incubation 10 min				
Peroxide	40	40	40	40
Incubation 10 min				
Working solution	100	100	100	100
Sodium acetate solution	10	10	10	10
The plate was vortexed, and the absorbance was measured at a wavelength of 570 nm.				

### The Cuvette method

The details of the procedure are shown in Table 3.

**Table 3.** The details of the protocol used to measure glutathione peroxidase activity.

Reagents	Test	Control	STD	Blank
Sodium phosphate buffer	1500	1900	1600	2000
Reduced glutathione (2 mM)	200		200	
Incubation 10 min				
Sample	100	100		
The reaction was initiated by adding peroxide:				
Peroxide	200		200	
Working solution	2000	2000	2000	2000
Sodium acetate solution	100	100	100	100
The test tubes were vortexed, and the absorbance was measured at a wavelength of 570 nm.				

### Calculation

One unit of GPx is defined as the enzyme activity at 25°C that can oxidize 1.0 µmol GSH to GSSG per minute at pH 7.0. Alternatively, it can be defined as the enzyme activity at 25°C that reduces 1.0 µmol H_2_O_2_ to H_2_O per minute at pH 7.0.

The test tube’s residual peroxide concentration = ($\frac{A\;\mathrm{Test}-A\;\mathrm{Control}}{A\;\mathrm{STD}}$) × Conc. of STD (1)

**Figure 1:**
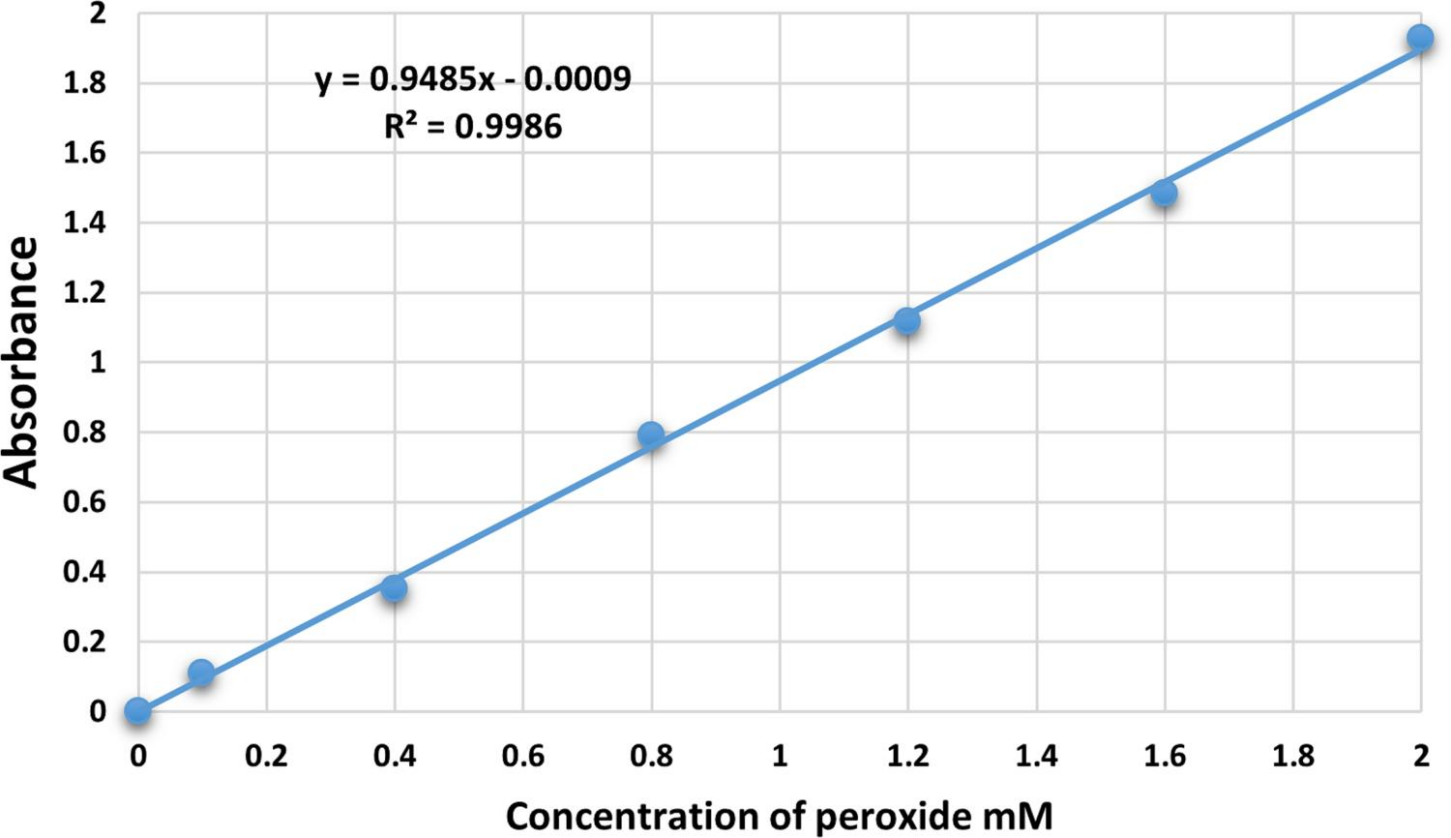
The standard curve of peroxide obtained by using the current protocol.

Additionally, the peroxide standard curve (Fig. 1) might be used to determine the remaining concentration of glutathione in the test tube. The number of micromoles of peroxide used is equal to the GPx activity.

Glutathione peroxidase activity (U/l) = ($\frac{C\mathrm{onc}.\;\mathrm{of}\;\mathrm{Peroxide}\;\mathrm{in}\;\mathrm{STD}-\mathrm{Conc}.\;\mathrm{of}\;\mathrm{residual}\;\mathrm{peroxide}\;\mathrm{in}\;\mathrm{test}\;\mathrm{tube}}{\mathrm{time}\;(10\;\min)}$)× ($\frac{\mathrm{Total}\;\mathrm{volume}\;(\mathrm{ml})}{\mathrm{Volume}\;\mathrm{of}\;\mathrm{the}\;\mathrm{sample}\;(\mathrm{ml})}$)× D.f. (2)

### The standard curve

To set up the assay’s standard curve, we diluted equivalent concentrations of peroxide standard with 0.1 M phosphate buffer at pH 7.0 to generate a range of standard concentrations from 0.1 to 2 mM H_2_O_2_. After mixing each tube of standards, we added 2000 µl of working solution, following the protocol in Table 1. We measured the absorbance against the blank after five minutes at 570 nm (as illustrated in Fig. 1).

### The method’s performance

The method’s execution followed bioanalytical validation guidelines by the Committee on Human Medicinal Products [26].

### Accuracy, selectivity, and reproducibility

To assess the accuracy and specificity of the new GPx procedure under conditions mimicking physiological interferences, an improved experimental setup was designed. Four test groups were prepared using 10 ml of 100 mM phosphate buffer solution (pH 7.4) as the base medium in separate vials. The first vial served as the control with only the buffer solution. The second vial was supplemented with a mixture of sugars (glucose, mannose, galactose, and ribose) at a concentration of 5 mM each to simulate carbohydrate interference. The third vial contained a mix of amino acids (isoleucine, leucine, aspartic acid, methionine, and valine) at 5 mM each to evaluate the impact of amino acid interference. The fourth vial included 1% bovine serum albumin (BSA) and 1% casein to represent protein interference. For the enzymatic reaction, 1 ml of GPx enzyme solution (250 U/l) was added to each vial, ensuring uniform mixing. The GPx activity was measured using the GPx Assay Kit (Catalog No: E-BC-K809-M) from Elabscience (China), which was calibrated to detect GPX activity. The recovery rate of the new GPx-Tiron method was calculated for each interfering condition by comparing the measured activity against the expected activity in the control. Additionally, the precision of the method was assessed by determining within-day and between-day repeatability using biological samples spiked with known GPx concentrations, with results expressed as relative standard deviation.

### Sensitivity and linearity

To evaluate the effectiveness of the new GPx-Tiron method, a range of GPx activity levels (5–500 IU/l) was tested for sensitivity and linearity. To measure linearity, the protocol was compared to the GPx-DTNB and GPx-CUPRAC methods to detect potential biases. The sensitivity of the modified GPx-Tiron method was evaluated by calculating the limits of quantification (LOQ) and limits of detection (LOD).

### Validation

A study was conducted to compare the GPx-Tiron method with the GPx-DTNB and GPx-CUPRAC methods. The GPx-DTNB and GPx-CUPRAC methods served as reference methods to ensure accuracy. GraphPad Prism 8.0 was utilized to analyze the results, estimate bias, and compare the analytical methods.

Bland–Altman plots were employed to determine the limits of agreement among the GPx-Tiron, GPx-DTNB, and GPx-CUPRAC methods. This analysis facilitated the evaluation of method parameters and confirmed that the variations between the techniques were normally distributed. The plots displayed an XY scatterplot, with the x-axis representing the absolute difference between the averages of the two methods and the y-axis representing the average calculated as (B–A). According to Bland and Altman, the mean difference within 95% of the confidence intervals is 1.96 standard deviations. Additionally, Passing–Bablok regression analysis was conducted to assess the agreement between the two analytical methodologies and identify any potential systematic bias.

## Results

### Tiron reagent as an appropriate probe for GPx activity measurement (the GPx-Tiron method)

Previous methods for measuring GPx activity have three significant drawbacks. First, using strong acids to stop enzymatic reactions presents considerable challenges. Second, measuring enzymatic activity is a time-consuming process. Third, adapting these methods for use with microplate readers is complicated due to the need for protein precipitation. To address these limitations, the current protocol employs a Tiron/ferrous ion (Fe^2+^) reagent, which eliminates the requirement for protein precipitation and effectively resolves the issues associated with earlier methods.

This new method presents a carefully designed protocol aimed at overcoming the limitations of traditional GPx measurement techniques. Central to this advancement is the innovative use of the Tiron/ferrous ion (Fe^2+^) reagent, which simplifies the assay by eliminating the need for protein precipitation, a step that previously introduced variability. By removing this step, the protocol also addresses the interference challenges that have hindered earlier GPx assays, significantly enhancing both precision and reliability.

A key feature of this protocol is its termination reagent, a carefully formulated mixture of ferrous ion (Fe^2+^) and Tiron (C_6_H_4_Na_2_O_8_S_2_). Upon addition, this reagent instantly stops GPx enzymatic activity, preventing the decomposition of H_2_O_2_ during the reaction. After GPx consumes part of the initial H_2_O_2_ substrate, the remaining H_2_O_2_ interacts with ferrous ions (Fe^2+^), proceeding through a classic Fenton-type redox reaction that oxidizes Fe^2+^ ions to ferric ions (Fe³^+^).

**Figure 2:**
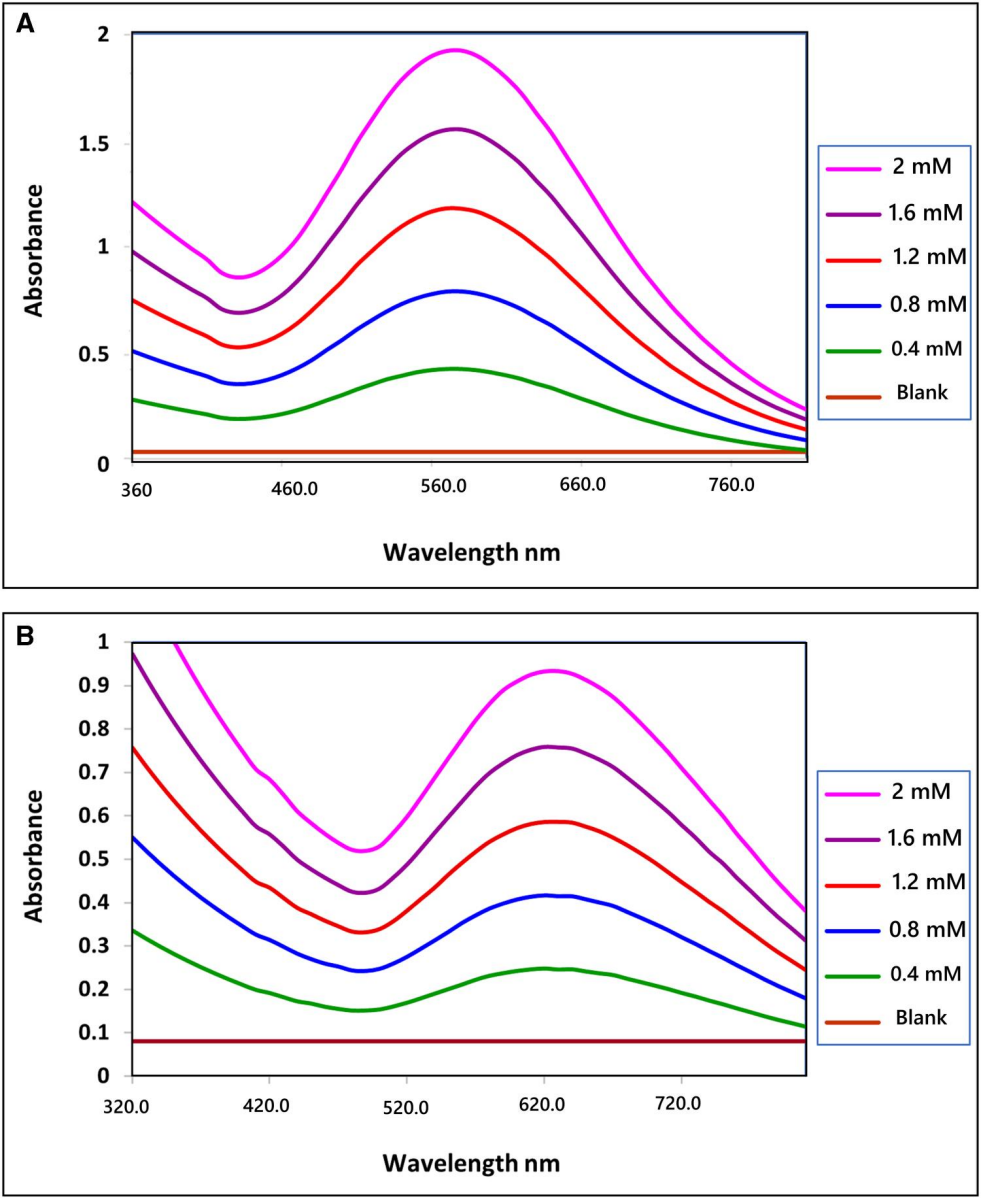
The absorbance of the ferri-Tiron complex inversely correlates with GPx enzyme activity. The spectrum exhibits a distinct peak at 570 nm, indicating the formation of the ferri-Tiron complex at pH ≤ 4.8, and a separate peak at 640 nm, signifying the formation of the complex at pH < 2. The H_2_O_2_ concentrations ranged from 0.4 mM to 2 mM.

**Scheme 1:**
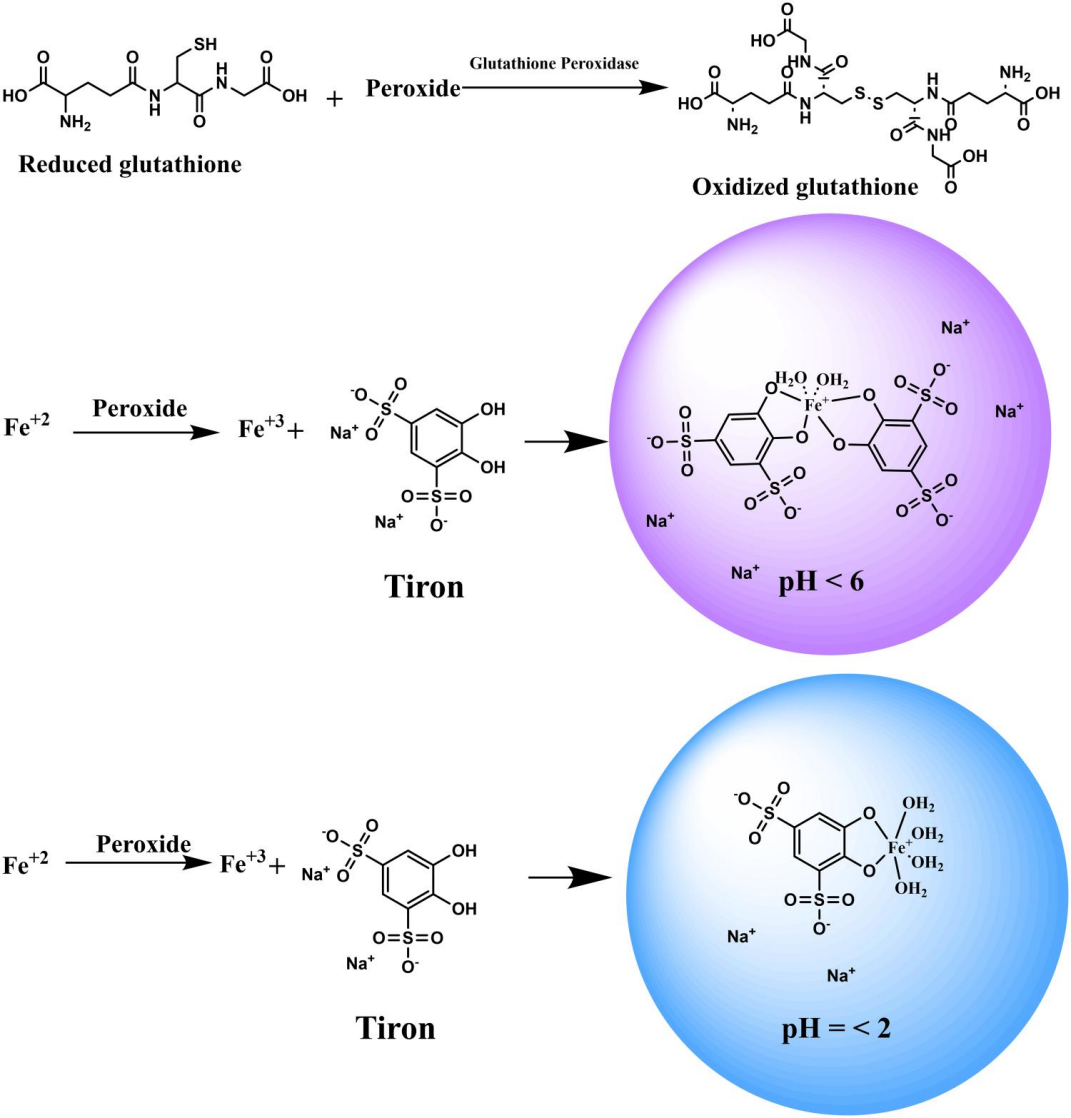
The GPx enzyme utilizes peroxide to transform the GSH into GSSG. Residual H_2_O_2_ undergoes Fenton-type redox reactions with Fe^2+^ ions, generating Fe³^+^ species that coordinate with Tiron through catechol moieties to form a stable ferri-Tiron complex [Fe(C_6_H_4_Na_2_O_8_S_2_)]³^+^. The assay is optimized at pH 4.8 to ensure complex stability and minimize interference from competing reactions.

The generated Fe³^+^ ions form a stable ferri-Tiron complex through specific coordination chemistry. The catechol moieties of Tiron bind tightly to Fe³^+^, resulting in a well-defined complex chemically represented as [Fe(C_6_H_4_Na_2_O_8_S_2_)]³^+^, as illustrated in Scheme 1. This formed complex exhibits unique absorbance characteristics that can be accurately measured using spectrophotometric techniques at a wavelength of 570 nm. Figure 2 shows a clear peak in the absorbance spectrum at 570 nm, which corresponds to the ferri-Tiron complex formed at pH ≤4.8, while it also shows a distinct peak at 640 nm, indicating the ferri-Tiron complex formed at pH ≤2. The absorbance at this wavelength is directly correlated with the residual concentration of H_2_O_2_, remaining after the GPx enzyme reactions. This finding confirms the assay’s ability to quantify GPx activity, as the absorbance of the ferri-Tiron complex is proportional to the amount of residual H_2_O_2_ present.

Notably, the stability and optical properties of the ferri-Tiron complex are strongly dependent on the pH of the reaction environment [27]. To maximize assay reliability, the protocol maintains a pH ≤4.8. Under these conditions, the colored complex remains stable over time. In contrast, at a neutral pH of 7.0, the complex may become unstable and be affected by other substances in the assay mixture, thereby compromising measurement accuracy.

The new GPx-Tiron method enables precise quantification of GPx activity, providing researchers with a reliable tool to investigate the enzyme’s critical role in cellular antioxidant defense mechanisms.

### Matrix effect

Matrix effects refer to the interference caused by sample components other than the target analyte during analytical measurements. In biological samples, endogenous peroxides and other reactive species can significantly interfere with GPx activity assays, potentially leading to inaccurate results. To address this challenge, the modified GPx-Tiron assay incorporates a systematic control strategy that effectively eliminates matrix interference. The experimental design includes parallel control tubes that contain all sample components except the GPx enzyme, allowing for precise identification and quantification of background interference. The absorbance readings from test tubes reflect contributions from both unreacted H_2_O_2_ (the desired signal) and various interfering substances present in biological samples. In contrast, control tube absorbance exclusively represents the interference from endogenous compounds, including native peroxides and other matrix components. By subtracting the control tube absorbance from the corresponding test tube absorbance, matrix-related interference is systematically eliminated. This differential measurement approach ensures that the final absorbance values accurately represent only the unreacted H_2_O_2_, providing reliable and contamination-free quantification of GPx enzymatic activity.

### Linearity and sensitivity

**Figure 3:**
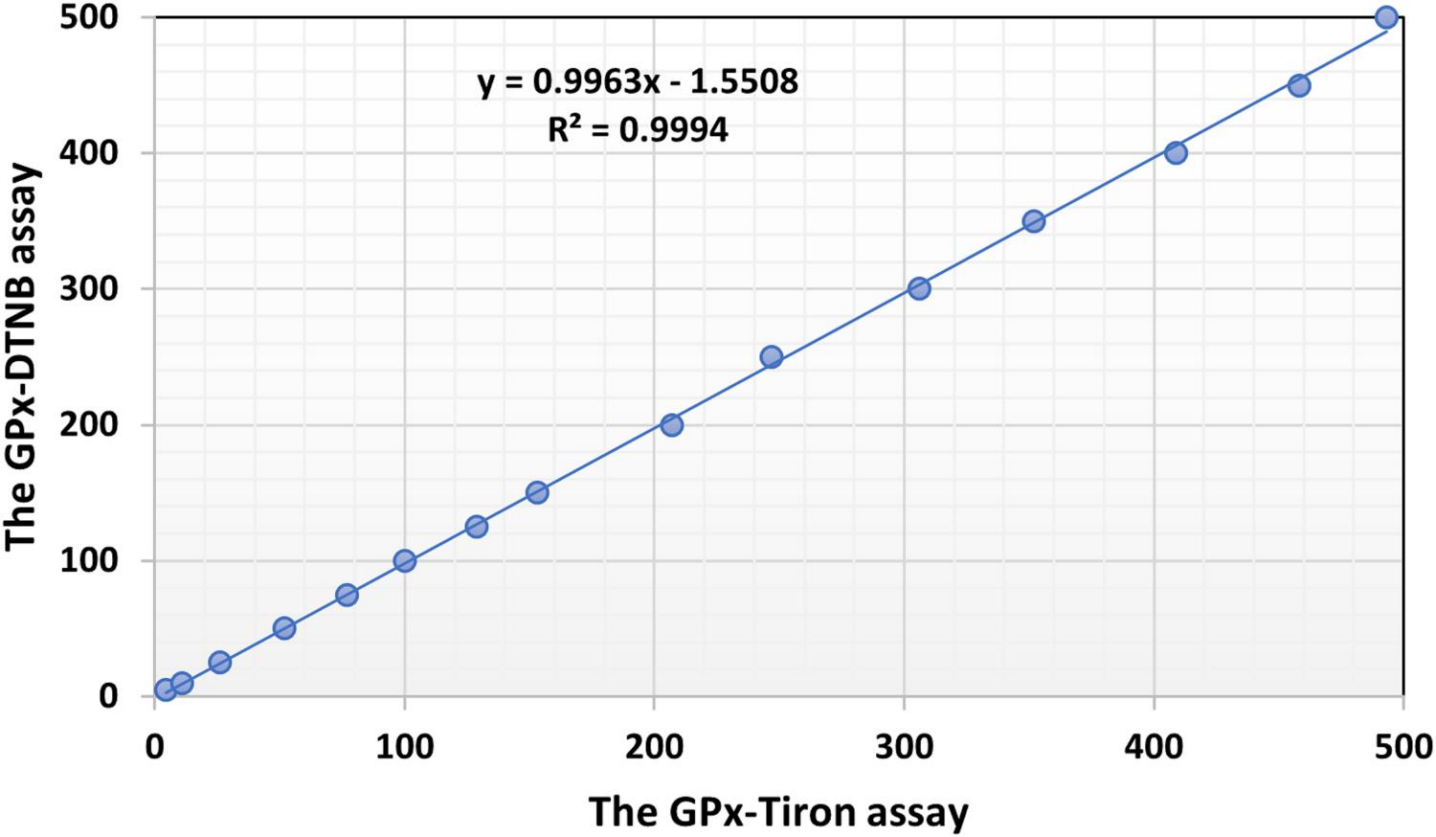
Linear response and comparative performance of the GPx-Tiron assay: validation against the GPx-DTNB reference method in biological samples.

Figure 3 illustrates that the new GPx-Tiron assay exhibits excellent analytical performance across a broad range of enzymatic activities. The method exhibits strict linearity over the concentration range of 5–500 U/l for GPx activity, with a good correlation coefficient (Pearson’s *r* = 0.999), confirming a robust linear relationship between enzyme concentration and measured response. The assay demonstrates high analytical sensitivity, with a LOD of 1 U/l and LOQ of 3 U/l. These low detection limits enable the accurate measurement of GPx activity even in samples with minimal enzyme concentrations, thereby expanding the method’s applicability to a diverse range of biological specimens.

Comparative analysis reveals that the linearity performance of the modified GPx-Tiron method matches that of the established reference GPx-DTNB assay, confirming its reliability as an alternative analytical method while offering the additional advantages of simplified sample preparation and enhanced stability.

### Accuracy, selectivity, and reproducibility of the new GPx-Tiron method

The modified GPx-Tiron assay demonstrates exceptional analytical specificity and high accuracy across various physiological interference conditions, including carbohydrates, amino acids, and proteins, as shown in Table 4. Recovery rates consistently range from 98.8% to 102.8%, indicating minimal matrix effects and robust performance in simulating complex biological environments. These results highlight the assay’s reliability in accurately measuring GPx activity at the expected concentration of 250 U/l, even in the presence of potential interfering biomolecules.

**Table 4.** The correlation between the relative percentage error of GPx activity and interfering biomolecules was determined using the new GPx-Tiron assay.

Flask no.	Contents	Added GPx (U/l)	Found GPx (U/l)	Recovery (%)
#1	Phosphate buffer (pH 7.0; 100 mM)	250	250	
#2	Glucose, mannose, galactose, and ribose (5 mM)	250	255	102
#3	Isoleucine, leucine, aspartic acid, methionine, and valine (5 mM)	250	257	102.8
#4	1% Bovine serum albumin (BSA) and 1% casein to represent protein	250	247	98.8

This outstanding specificity and minimal interference eliminate the need for extensive sample preparation, yielding practical advantages such as faster processing times, reduced reagent consumption, lower risk of analyte loss, and improved laboratory workflow efficiency. Consequently, the GPx-Tiron method is particularly well-suited for high-throughput applications where minimizing sample preparation complexity and processing duration is critical.

### Validation and method comparison

The accuracy and reliability of the novel GPx-Tiron assay were rigorously assessed using two established statistical methodologies: Bland–Altman plot analysis and Passing–Bablok regression. All statistical computations were meticulously performed using GraphPad Prism software (GraphPad Software, San Diego, CA, USA). In this comprehensive evaluation, the performance of the GPx-Tiron method was directly and systematically compared against two widely recognized reference assays: the traditional GPx-DTNB method and the GPx-CUPRAC method. To ensure a robust and representative evaluation of the assay’s accuracy and reliability, enzyme samples were prepared through serial dilutions, yielding glutathione peroxidase (GPx) activity levels that spanned a broad range from 50 to 500 U/l, allowing for a direct paired comparison.

**Figure 4:**
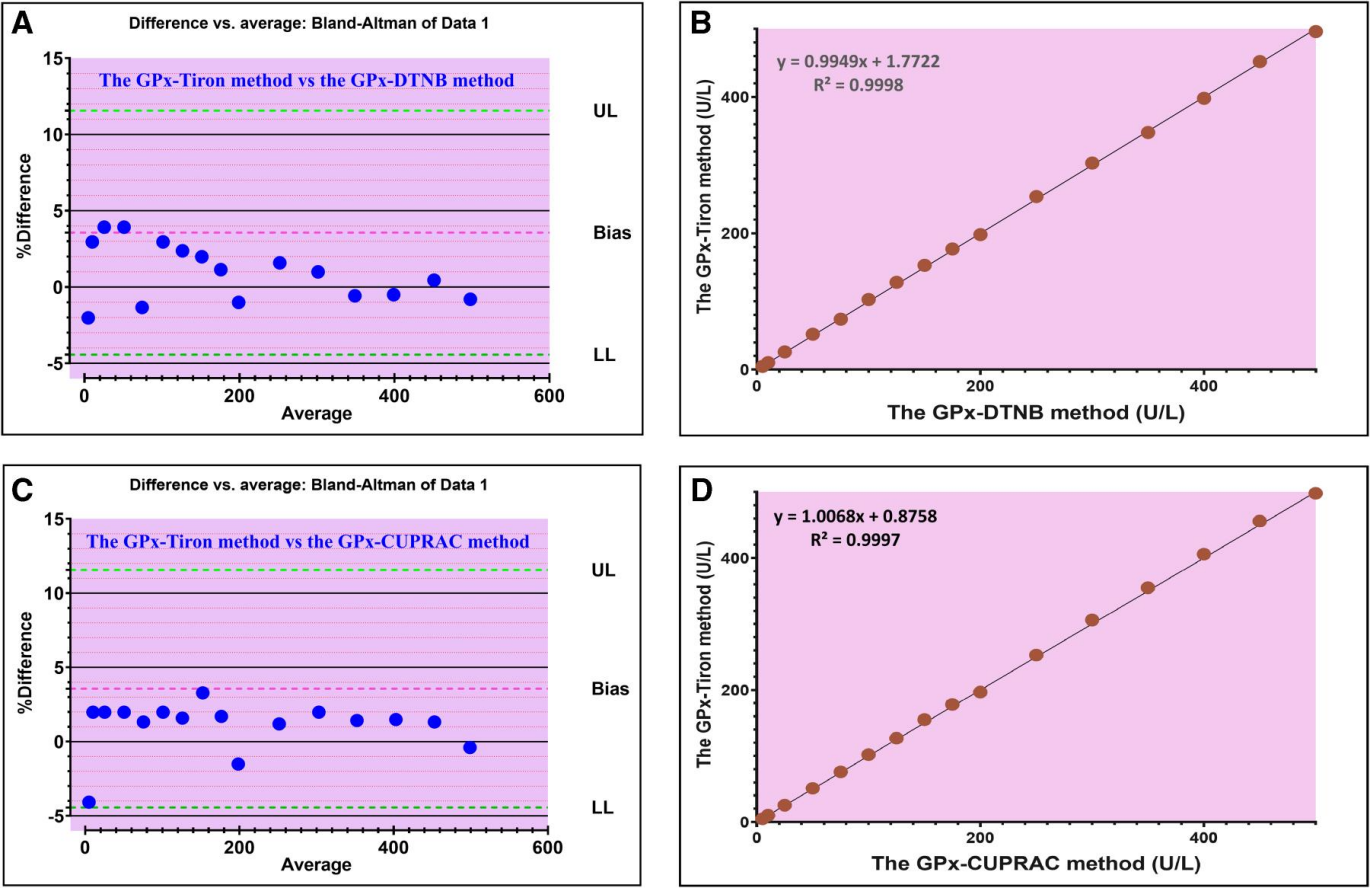
(A) Bland–Altman plot assessing agreement between the GPx-Tiron method and the GPx-DTNB method. (B) Bland–Altman plot assessing agreement between the GPx-Tiron method and the GPx-CUPRAC method. (C) Passing–Bablok regression analysis comparing the GPx-Tiron method and the GPx-DTNB method. (D) Passing–Bablok regression analysis comparing the GPx-Tiron method and the GPx-CUPRAC method.

Figure 4A and C visually present the Bland–Altman plots, which graphically illustrate the agreement between the GPx-Tiron method and the GPx-DTNB and GPx-CUPRAC reference methods, respectively. In these plots, the Y-axis represents the percentage difference between paired measurements obtained from the two methods, while the x-axis displays the average value of these measurements. This crucial visualization technique facilitates the assessment of systematic bias and defines the limits of agreement, thereby enabling a robust evaluation of the new assay against established standards. A slight mean bias, coupled with narrow limits of agreement, as demonstrably observed in our analysis, serves as strong evidence indicating that the GPx-Tiron method possesses high accuracy and reliability, confirming its comparability to the reference methods.

Figure 4B further details the relationship between the GPx-Tiron and GPx-DTNB methods through a scatter plot, which highlights a remarkably strong positive correlation, as evidenced by a correlation coefficient (r) of 0.99. Subsequent linear regression analysis of these data points yielded the equation y = 0.9949x + 1.7722, where “y” denotes the GPx activity measured by the GPx-Tiron method and “x” signifies the GPx activity determined by the GPx-DTNB method. The regression slope of 0.9949, being exceptionally close to unity (1.0), along with a minimal y-intercept of 1.7722, collectively demonstrates an almost perfect agreement and negligible systematic bias between these two assays.

In a parallel comparison, Fig. 4D illustrates the concordance between the GPx-Tiron and GPx-CUPRAC methods, similarly exhibiting a strong correlation with a coefficient (*r*) of 0.99. The linear regression analysis for this comparison resulted in the equation *y* = 1.0068x + 0.8758. Once again, the derived slope of 1.0068, which closely approximates unity, and the small intercept of 0.8758 provide emphatic confirmation of the high precision and accuracy of the GPx-Tiron method when validated against the GPx-CUPRAC reference standard. These compelling statistical findings underscore the robustness and reliability of the GPx-Tiron assay across various analytical contexts.

### Application #1

The study employed a new method of the GPx assay to meticulously evaluate GPx activity in homogenates derived from various animal tissues. As detailed in Table 5, the results revealed a marked increase in GPx activity within liver tissue homogenates, effectively validating the accuracy and reliability of this innovative GPx-Tiron method.
It was noted that GPx activity varies significantly based on the type of sample and the substrate used. Specifically, the highest levels of activity were found in erythrocytes, followed by liver, kidney, and serum samples. This variability underscores the importance of sample selection in GPx activity assessments. Furthermore, the modified GPx-Tiron assay demonstrated exceptional reproducibility, producing consistent results both within the same day and over multiple days, thereby confirming its robustness and reliability for future biochemical research applications.

**Table 5.** Comparative analysis of GPx activity in rat tissue homogenates using the modified GPx-Tiron method and reference GPx-DTNB assay.

Samples	Peroxides	The new GPx-Tiron method	The GPx-DTNB method
		Mean ± standard deviation (SD)	Mean ± SD
Serum (U. l^−1^)	H_2_O_2_	418 ± 6	411 ± 7
	tBuOOH	387 ± 5	379 ± 6
	CuOOH	402 ± 6	396 ± 7
Erythrocytes GPx (U/g.Hb)	H_2_O_2_	227 ± 4	223 ± 5
	tBuOOH	198 ± 3	194 ± 4
	CuOOH	211 ± 4	209 ± 5
Kidney GPx (U/mg. protein)	H_2_O_2_	152 ± 3	146 ± 4
	tBuOOH	130 ± 2	127 ± 3
	CuOOH	142 ± 3	137 ± 4
Liver (U/mg. protein)	H_2_O_2_	320 ± 5	315 ± 6
	tBuOOH	293 ± 4	288 ± 5
	CuOOH	308 ± 5	298 ± 6

Assessment of glutathione peroxidase activity in liver tissue is a well-established method for evaluating hepatic antioxidant defense mechanisms and resistance to oxidative stress. Comparative studies across various laboratory animal species have demonstrated the utility of hepatic GPx measurements in characterizing tissue-specific antioxidant capacity and susceptibility to oxidative damage.

### Properties of the GPx-Tiron method

The measurement of GPx activity is crucial for understanding its role in cellular antioxidant defense mechanisms. Traditional methods, such as the GPx-DTNB assay, have been widely used but are often hindered by significant drawbacks, including the need for protein precipitation, susceptibility to interference from various biological components, and lengthy processing times. These limitations can compromise the accuracy and reliability of GPx activity measurements, particularly in complex biological samples.

In response to these challenges, the modified GPx-Tiron assay was developed, utilizing Tiron as a probe and a novel termination reagent composed of ferrous ammonium sulfate (FAS). This innovative approach effectively halts the enzymatic activity of GPx, allowing for accurate quantification of residual H_2_O_2_ through the formation of a stable ferri-Tiron complex. The assay is optimized to operate at an acidic pH of 5.0, which enhances the stability of the colored complex and minimizes interference from competing reactions.

The results from the new GPx-Tiron method demonstrate exceptional analytical performance, with a linear response over a wide range of GPx activity levels (5–500 U/l) and high sensitivity, evidenced by low LOD and LOQ. The method’s robustness is further validated through comparative analyses with established reference assays, such as GPx-DTNB and GPx-CUPRAC, using statistical methodologies like Bland–Altman plots and Passing–Bablok regression. These analyses reveal a strong correlation between the modified GPx-Tiron method and the reference methods, indicating minimal systematic bias and high accuracy.

Moreover, the new assay exhibits remarkable specificity, with recovery rates consistently ranging from 98.8% to 102.8% across various physiological interference conditions, including carbohydrates, amino acids, and proteins. This reliability is crucial for high-throughput applications in both research and clinical diagnostics, where minimizing sample preparation complexity and processing duration is essential.

## Conclusion

The modified GPx-Tiron assay represents a significant advancement in measuring glutathione peroxidase activity, addressing the limitations of traditional methodologies. By eliminating the need for protein precipitation and enhancing the assay’s specificity and sensitivity, this innovative approach provides a reliable and efficient tool for both researchers and clinicians. The assay’s ability to deliver accurate results in the presence of potential interfering substances underscores its practical utility in diverse analytical settings. As such, the GPx-Tiron method stands as a valuable alternative for assessing oxidative stress and evaluating antioxidant capacity in various biological specimens, paving the way for further research into the critical role of GPx in health and disease.

## Data Availability

The authors declare that all data supporting this study’s findings are included in the article. Further data are available from the corresponding author upon request.
